# Positive correlation between fatty liver index and hyperuricemia in hypertensive Chinese adults: a H-type hypertension registry study

**DOI:** 10.3389/fendo.2023.1183666

**Published:** 2023-06-02

**Authors:** Chao Yu, Xinlei Zhou, Tao Wang, Lingjuan Zhu, Wei Zhou, Huihui Bao, Xiaoshu Cheng

**Affiliations:** ^1^ Center for Prevention and Treatment of Cardiovascular Diseases, the Second Affiliated Hospital of Nanchang University, Nanchang, Jiangxi, China; ^2^ Department of Cardiovascular Medicine, the Second Affiliated Hospital of Nanchang University, Nanchang, Jiangxi, China; ^3^ Jiangxi Provincial Cardiovascular Disease Clinical Medical Research Center, Nanchang, Jiangxi, China

**Keywords:** fatty liver index, hyperuricemia, hypertension, sex differences, non-alcoholic fatty liver disease

## Abstract

**Background:**

Few studies have examined the relationship between fatty liver index (FLI) and hyperuricemia (HUA). This study explores the relationship between FLI and HUA in hypertensive patients.

**Methods:**

A total of 13,716 hypertensive subjects were included in the current study. FLI, a simple index calculated from triglycerides (TG), waist circumference (WC), body mass index (BMI), and γ -glutamyltransferase (GGT), was used as a useful predictor of nonalcoholic fatty liver disease (NAFLD) distribution. HUA was defined as serum uric acid ≥ 360 μmol/L for females and ≥ 420 μmol/L for males.

**Results:**

The mean value of total FLI was 31.8 ± 25.1. Multiple logistic analyses revealed a significant positive correlation between FLI and HUA (OR, 1.78; 95% CI: 1.69–1.87). A subgroup analysis demonstrated that the correlation between FLI (< 30 vs. ≥ 30) and HUA was significant in both sexes (P for interaction = 0.006). Further analyses stratified by sex indicated a positive correlation between FLI and HUA prevalence among male and female subjects. However, the correlation between FLI and HUA was stronger in female subjects than in males (male: OR, 1.70; 95% CI: 1.58–1.83; female: 1.85; 95% CI: 1.73–1.98).

**Conclusion:**

This study demonstrates a positive correlation between FLI and HUA in hypertensive adults, but stronger in females than males.

## Highlight

- Fatty liver index (FLI) has a positive correlation with hyperuricemia (HUA) in hypertensive adults, but stronger in females than males.

## Introduction

Hyperuricemia (HUA) is a metabolic disease caused by abnormal purine metabolism resulting in overproduction of uric acid (primarily in liver) and its decreased renal and intestinal excretion (1). HUA is the main risk factor for gout, a very painful long-term systemic inflammatory arthritis caused by the deposition of monosodium urate (MSU) crystal (2, 3). A systematic review and meta-analysis revealed a pooled HUA prevalence of 13.3% between 2000 and 2014 in mainland China, with a higher prevalence among males than females (4). Moreover, numerous studies have discovered that HUA prevalence is typically higher in high-income countries than in economically developing countries (4–8). Additionally, gout and HUA prevalence has increased with age, posing a huge burden for aging countries (9). Gout and HUA can cause $116 million in socio-economic losses yearly in the United States (10). Previous studies have stated association of HUA with hypertension (11, 12), adverse cardiovascular outcomes (13), stroke (14), metabolic syndrome (15), and chronic kidney disease (16). Particularly, HUA has been correlated with the severity of hypertension (14). Therefore, the study of HUA in hypertensive populations can help to optimize the risk stratification of HUA and provide important clinical guidance for the future personalized care of hypertensive individuals. Recent studies suggest that non-alcoholic fatty liver disease (NAFLD) may be an independent risk factor for HUA and is associated with its occurrence and development (17). Additionally, HUA occurrence and development may vary according to gender (18, 19).

Fat accumulation in the liver due to metabolic disorders causes NAFLD (20). It has also been linked to hypertension, insulin resistance, and metabolic syndrome (19). Previous studies have reported that NAFLD patients have significantly higher Serum urate levels than normal subjects. NAFLD presence also significantly increases HUA risk (17, 21) due to the increased xanthine oxidase (XO) expression or activity in NAFLD patients, which catalyzes uric acid production (17). Additionally, there is a sex difference in NAFLD (22), with females having a worse prognosis for HUA than males (19). Thus, sex may serve as a mediator in the association between NAFLD and HUA (21, 23, 24).

Abdominal ultrasonography and liver biopsies are the gold standard for diagnosing NAFLD, but they are an invasive procedure with elevated risks and economic costs that preclude large-scale epidemiological studies. Therefore, Bedogni et al. (25) developed a compound index that includes triglycerides (TG), waist circumference (WC), body mass index (BMI), and γ-glutamyltransferase (GGT) to evaluate NAFLD, namely fatty liver index (FLI). FLI has an overall accuracy of 84% in detecting NAFLD (26); thus, FLI can be used as an alternative predictor of NAFLD. Moreover, FLI has outstanding application value in large-scale epidemiological studies. FLI has been used in numerous scientific investigations with large sample sizes (27–29). A significant association has been reported between FLI and the risk of HUA (30). However, the study included only 1,284 subjects, the sample was small, and several limitations like the study is cross-sectional in nature with possible Neyman bias, type II error, and urate-lowering therapy dietary habits not adjusted etc.

This study aims to address the above knowledge gap and explore the correlation between FLI and HUA in hypertensive patients using China H-type Hypertension Registry Study data. This study also examines sex difference in this association and further explores potential modifiers between them.

## Methods

### Study population

The data from the China H-type Hypertension Registry Study (registration number: ChiCTR1800017274) were analyzed in this study. It was an ongoing observational study of the real world in Wuyuan, China, from March 2018 to August 2018. The study design and methods have been described in detail previously (31, 32). Hypertension patients over 18 years old were included. Participants who could not sign informed consent due to psychological or nervous system injury and could not have long-term follow-up according to the study protocol were excluded. The study was approved by the ethics committee of the Institute of Biomedicine, Anhui Medical University (NO. CH1059), and the Second Affiliated Hospital of Nanchang University (NO. 2018019). Additionally, an informed consent form was signed by each participant.

A total of 14,234 hypertensive subjects met the inclusion and exclusion criteria. Participants who lost FLI data (n = 12), Serum urate data (n = 0), or using lipid-lowering medications (n = 506) were excluded from our analysis. The analysis involved 13,716 participants. The exclusion process details are described in **Figure S1**.

### Data collection

The study population’s demographic and behavioral characteristics were collected through a health interview conducted by trained medical staff using a validated questionnaire. This demographic and behavioral data included age, sex, education level (Illiteracy, primary, and at least secondary), physical activity (mild, moderate, and vigorous), smoking and drinking status, diabetes history, stroke history, and medication information (antihypertensive drugs, lipoprotein-lowering drugs, and glucose-lowering drugs). Current smoking was defined as smoking ≥ 1 cigarette per day for one year or more or a cumulative smoking amount of ≥ 360 cigarettes per year.

Anthropometric data, body height, WC, measured to the nearest 5 mm directly touching the participant’s skin using cloth tape. Blood pressure (BP) was assessed by trained medical staff to limit interobserver variability in measurement. After 5 min of rest as seated BP was measured using an electronic sphygmomanometer (Omron; Dalian, China), following the standard method and appropriately sized cuffs. Three measurements were performed on the right arm, with 1 min intervals between them, and the mean value was calculated. BMI was defined as body weight/hei^2^ (kg/m^2^).

Fasting blood samples were obtained from all of the patients. All the biochemical measurements were conducted at the Biaojia Biotechnology, Shenzhen, Guangdong Province, China using automatic clinical analyzers (Beckman Coulter, USA). Biochemical data, including fasting plasma glucose (FPG), homocysteine (Hcy), TG, total cholesterol (TC), high-density lipoprotein cholesterol (HDL-C), low-density lipoprotein cholesterol (LDL-C), aspartate aminotransferase, alanine aminotransferase, and GGT, were obtained from the fasting blood samples. The estimated glomerular filtration rate (eGFR) was calculated using the Chronic Kidney Disease Epidemiology Collaboration (CKD-EPI) equation (33).

### Definition of the FLI and HUA

FLI was calculated using Bedogni et al. (25) method with following formula: FLI = (*e* ^0.953×ln(TG)+0.139×BMI+0.718×ln (GGT) + 0.053 ×WC−15.745^)/(1+ *e* ^0.953×ln(TG)+0.139×BMI+0.718×ln (GGT) + 0.053 ×WC−15.745^)×100. In the above formula, the unit of BMI, WC, TG, and GGT were kg/m^2^, cm, mmol/L, and U/L, respectively. FLI scores ranged from 0 to 100. Since FLI ≥ 30 was suggested as a cutoff level to rule in hepatic steatosis (25). According to clinical significance, FLI was divided into two groups (< 30, ≥ 30). HUA was defined as serum uric acid ≥ 360 μmol/L for females and ≥ 420 μmol/L for males (34).

### Other definition

Diabetes mellitus was defined as self-reported physician diagnosis, FBG concentration ≥ 7.0 mmol/L, or use of glucose-lowering drugs. The medical history of the stroke was a self-reported stroke that was primarily collected *via* a questionnaire.

### Statistical analysis

Participants’ baseline characteristics were presented as mean ± standard deviation (SD) for continuous variables and as a percentage (%) for categorical variables using an FLI clinical cutoff. Accordingly, differences in population characteristics by the clinical cutoff of FLI were compared using mono factorial variance test, or *χ^2^* tests.

FLI was divided into two groups: normal group FLI < 30 and hepatic steatosis group FLI ≥ 30, for analysis based on its clinical cutoff. The FLI was assessed by two groups and continuous variables. Multivariate logistic regression models were used to evaluate the association between the FLI and HUA in hypertensive participants of both sexes. Covariates were included as potential confounders in the final multivariate logistic regression models if they changed the estimates of FLI on HUA by more than 10% (35) or were known as traditional risk factors for HUA. Four multivariate regression models were considered: Model 1: age and sex (only for the overall population); Model 2: age, sex, BMI, WC, education, living standard, physical activity, current smoking, current drinking, Hcy, creatinine, and eGFR; Model 3: age, sex (only for overall population), BMI, WC, education, living standard, physical activity, current smoking, current drinking, Hcy, creatinine, eGFR, diabetes mellitus, antihypertensive drugs, and glucose-lowering drugs. A generalized additive model and a fitted smoothing curve (penalized spline method) were utilized to assess the dose-response association between FLI and HUA prevalence. Stratification analyses were performed based on age (< 65 vs. ≥ 65y), living standard (preferably, commonly, and poor), current smoking (no vs. yes), current drinking (no vs. yes), eGFR (< 60 vs. ≥ 60 mL/min/1.73 m^2^), diabetes mellitus (no vs. yes), LDL-C (<1.8 vs. ≥1.8 mmol/L), HDL-C (<1.0 vs. ≥1.0 mmol/L), and Hcy (<15 vs. ≥15 μmol/L) to test whether these factors could modify the association between FLI and HUA prevalence in different sex. These were tested by adding a cross-product term between covariates and FLI to the model.

Statistical analyses were conducted using the statistical packages R, version 4.2.3, (R Foundation for Statistical Computing; http://www.r-project.org). A two-sided P-value < 0.05 was considered statistically significant in all analyses.

## Results

### Study participants and baseline characteristics

This study enrolled 13,716 hypertensive subjects (mean age 63.8 ± 9.4 years old, 47.2% male subjects). The average FLI was 31.8 ± 25.1, with HUA accounting for 44.5%. **Table 1** presents the baseline characteristics of the participants according to FLI (< 30 vs. ≥ 30) in both sexes. In males, higher FLI was associated with higher BMI, WC, GGT, TC, TG, LDL-C, and UA but lower levels of Hcy and HDL-C. They were also more likely to be people with elevated living standards, current drinkers, HUA, diabetes, using glucose-lowering drugs, and younger adults. The baseline characteristics of the female subjects were identical to those of the males. Hcy levels, living standards, and alcohol consumption did not differ significantly between the two groups (P > 0.05). Patients with a higher FLI were more likely to use antihypertensive drugs. **Table S1** displays the participants’ baseline characteristics based on FLI classification (< 30, ≥ 30).

**Table 1:** Baseline characteristics of different sex participants according to FLI.

Characteristics	Total	Males				Females			
		FLI clinical cutoff				FLI clinical cutoff			
		<30	>=30	SD	P-value	<30	>=30	SD	P-value
Participants	13716	3581	2897			4139	3099		
Age, year	63.8 ± 9.4	66.8 ± 9.0	60.0 ± 9.5	0.7 (0.7, 0.8)	<0.001	64.9 ± 9.3	62.3 ± 8.4	0.3 (0.2, 0.3)	<0.001
BMI, kg/m^2^	23.6 ± 3.7	21.2 ± 2.4	26.0 ± 3.8	1.5 (1.5, 1.6)	<0.001	21.7 ± 2.5	26.5 ± 3.0	1.7 (1.7, 1.8)	<0.001
WC, cm	83.7 ± 9.9	78.0 ± 7.2	91.9 ± 7.1	1.9 (1.9, 2.0)	<0.001	77.7 ± 7.3	90.8 ± 7.4	1.8 (1.7, 1.8)	<0.001
Education, n (%)				0.5 (0.4, 0.5)	<0.001			0.4 (0.4, 0.5)	0.004
Illiteracy	4255 (38.3)	570 (19.1)	224 (9.5)			2040 (62.0)	1421 (57.7)		
Primary	4650 (41.9)	1666 (55.9)	1101 (46.5)			1034 (31.4)	849 (34.5)		
Secondary and above	2197 (19.8)	745 (25.0)	1043 (44.0)			218 (6.6)	191 (7.8)		
Living standard, n (%)				0.2 (0.1, 0.2)	<0.001			0.1 (-0.0, 0.1)	0.153
preferably	1456 (13.1)	372 (12.5)	396 (16.7)			410 (12.5)	278 (11.3)		
commonly	7458 (67.2)	1983 (66.5)	1600 (67.6)			2184 (66.3)	1691 (68.7)		
poor	2188 (19.7)	626 (21.0)	372 (15.7)			698 (21.2)	492 (20.0)		
Physical activity ^a^, n (%)				0.1 (0.0, 0.1)	0.107			0.1 (0.0, 0.1)	0.076
Mild	6203 (55.9)	1632 (54.7)	1311 (55.4)			1825 (55.4)	1435 (58.3)		
Moderate	2589 (23.3)	710 (23.8)	601 (25.4)			744 (22.6)	534 (21.7)		
Vigorous	2310 (20.8)	639 (21.4)	456 (19.3)			723 (22.0)	492 (20.0)		
Current smoking, n (%)	3564 (26.0)	1846 (51.6)	1319 (45.5)	0.1 (0.1, 0.2)	<0.001	258 (6.2)	141 (4.6)	0.1 (0.0, 0.1)	0.002
Current alcohol drinking, n (%)	3005 (21.9)	1328 (37.1)	1294 (44.7)	0.2 (0.1, 0.2)	<0.001	226 (5.5)	157 (5.1)	0.0 (-0.0, 0.1)	0.463
GGT, U/L	33.2 ± 43.2	25.4 ± 23.1	60.4 ± 70.1	0.7 (0.6, 0.7)	<0.001	17.7 ± 11.0	37.4 ± 42.1	0.6 (0.6, 0.7)	<0.001
TC, mmol/L	5.2 ± 1.1	4.8 ± 1.0	5.2 ± 1.1	0.4 (0.3, 0.4)	<0.001	5.3 ± 1.1	5.5 ± 1.2	0.2 (0.2, 0.3)	<0.001
TG, mmol/L	1.8 ± 1.2	1.1 ± 0.5	2.4 ± 1.5	1.1 (1.1, 1.2)	<0.001	1.4 ± 0.6	2.6 ± 1.5	1.0 (0.9, 1.0)	<0.001
LDL-C, mmol/L	3.0 ± 0.8	2.7 ± 0.7	3.1 ± 0.8	0.6 (0.6, 0.7)	<0.001	3.0 ± 0.8	3.3 ± 0.8	0.4 (0.4, 0.5)	<0.001
HDL-C, mmol/L	1.6 ± 0.4	1.6 ± 0.4	1.4 ± 0.4	0.6 (0.6, 0.7)	<0.001	1.7 ± 0.4	1.5 ± 0.4	0.5 (0.4, 0.5)	<0.001
Hcy,μmol/L	18.0 ± 11.0	20.8 ± 13.0	20.2 ± 14.5	0.0 (-0.0, 0.1)	<0.001	15.8 ± 7.4	15.6 ± 7.1	0.0 (-0.0, 0.1)	0.494
Creatinine, μmol/L	72.7 ± 46.4	86.5 ± 56.7	83.2 ± 41.7	0.1 (0.0, 0.1)	0.834	61.9 ± 45.1	61.4 ± 29.9	0.0 (-0.0, 0.1)	0.007
eGFR, mL/min/1.73 m^2^	88.3 ± 20.2	83.6 ± 20.7	88.7 ± 19.7	0.3 (0.2, 0.3)	<0.001	90.1 ± 19.7	91.0 ± 19.8	0.0 (-0.0, 0.1)	0.023
Serum urate, μmol/L	419.1 ± 120.7	443.9 ± 112.4	492.7 ± 121.4	0.4 (0.4, 0.5)	<0.001	354.5 ± 98.1	408.1 ± 108.9	0.5 (0.5, 0.6)	<0.001
Hyperuricemia, n (%)	6099 (44.5)	1906 (53.2)	2048 (70.7)	0.4 (0.3, 0.4)	<0.001	886 (21.4)	1259 (40.6)	0.4 (0.4, 0.5)	<0.001
Diabetes mellitus^b^, n (%)	2436 (17.8)	333 (9.3)	674 (23.3)	0.4 (0.3, 0.4)	<0.001	565 (13.7)	864 (27.9)	0.4 (0.3, 0.4)	<0.001
Antihypertensive drugs, n (%)	8781 (64.0)	2261 (63.2)	1870 (64.5)	0.0 (-0.0, 0.1)	0.246	2547 (61.5)	2103 (67.9)	0.1 (0.1, 0.2)	<0.001
Glucose-lowering drugs, n (%)	661 (4.8)	85 (2.4)	171 (5.9)	0.2 (0.1, 0.2)	<0.001	154 (3.7)	251 (8.1)	0.2 (0.1, 0.2)	<0.001
FLI	31.8 ± 25.1	12.7 ± 7.9	57.7 ± 18.9	3.1 (3.0, 3.2)	<0.001	13.5 ± 7.9	54.0 ± 17.3	3.0 (3.0, 3.1)	<0.001

SD, Standardize difference; FLI, Fatty Liver Index; BMI, body mass index; WC, waist circumference; DBP, diastolic blood pressure; GGT, γ-glutamyltransferase; TC, total cholesterol; TG, triglycerides; HDL-C, high-density lipoprotein cholesterol; LDL-C, low-density lipoprotein cholesterol; Hcy, homocysteine; eGFR, estimated glomerular filtration rate.

a Physical activity was defined as mild, moderate, or vigorous according to the participant’s personal evaluation.

b diabetes mellitus was defined as self-reported physician diagnosis of diabetes or FBG concentration ≥ 7.0 mmol/L or use of glucose-lowering drugs.

### Association of FLI with HUA

**Figure 1:**
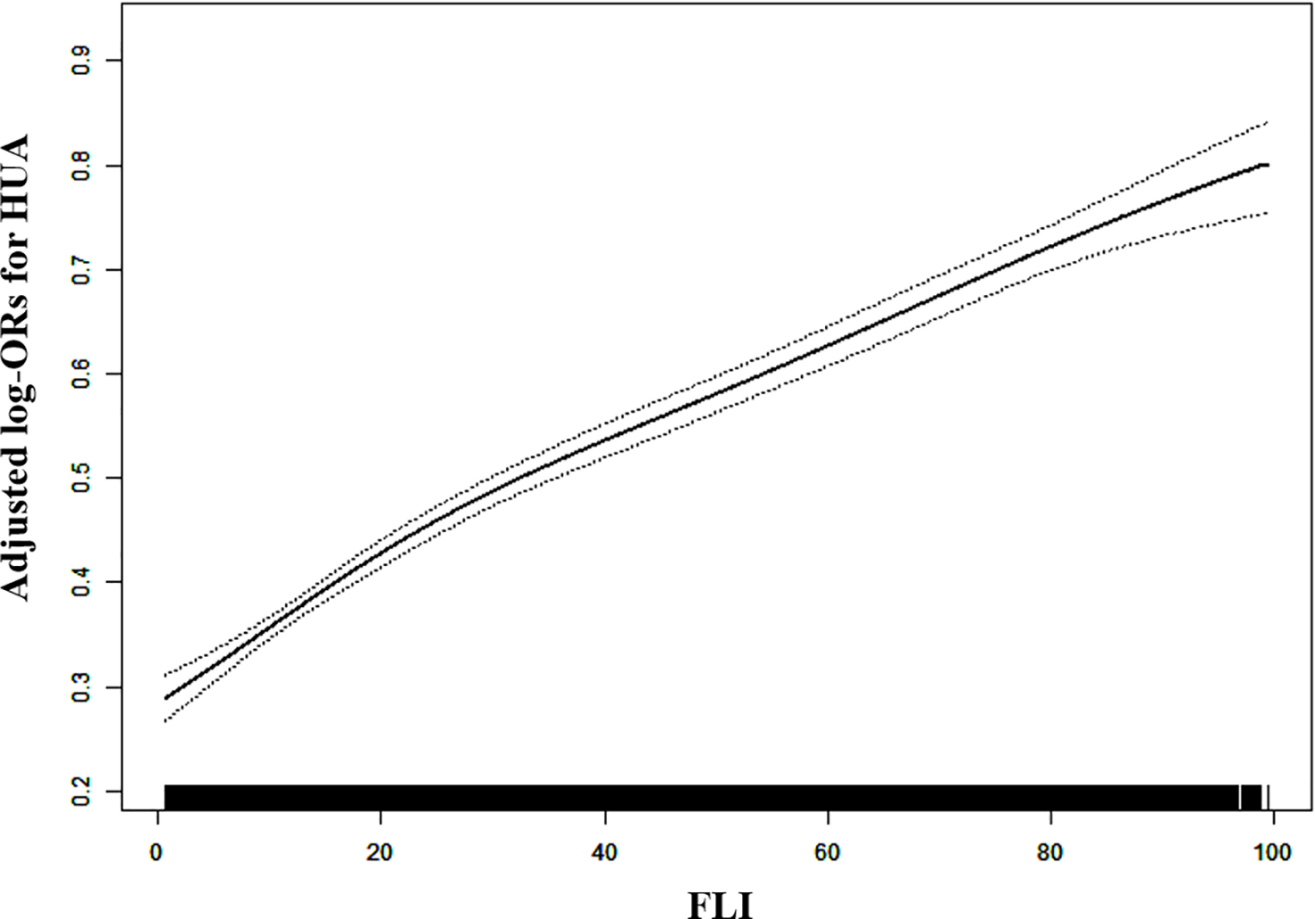
Dose-response association between FLI and HUA prevalence in hypertensive Chinese patients. A linear correlation between FLI and HUA prevalence was found (P < 0.05). The solid and dashed lines represent the estimated values and corresponding 95% confidence interval, respectively. Adjustment factors included age, sex, BMI, WC, Education, Living standard, Physical activity, current smoking, current drinking, Hcy, creatinine, eGFR, diabetes mellitus, antihypertensive drugs, and glucose-lowering drugs.

**Figure 2:**
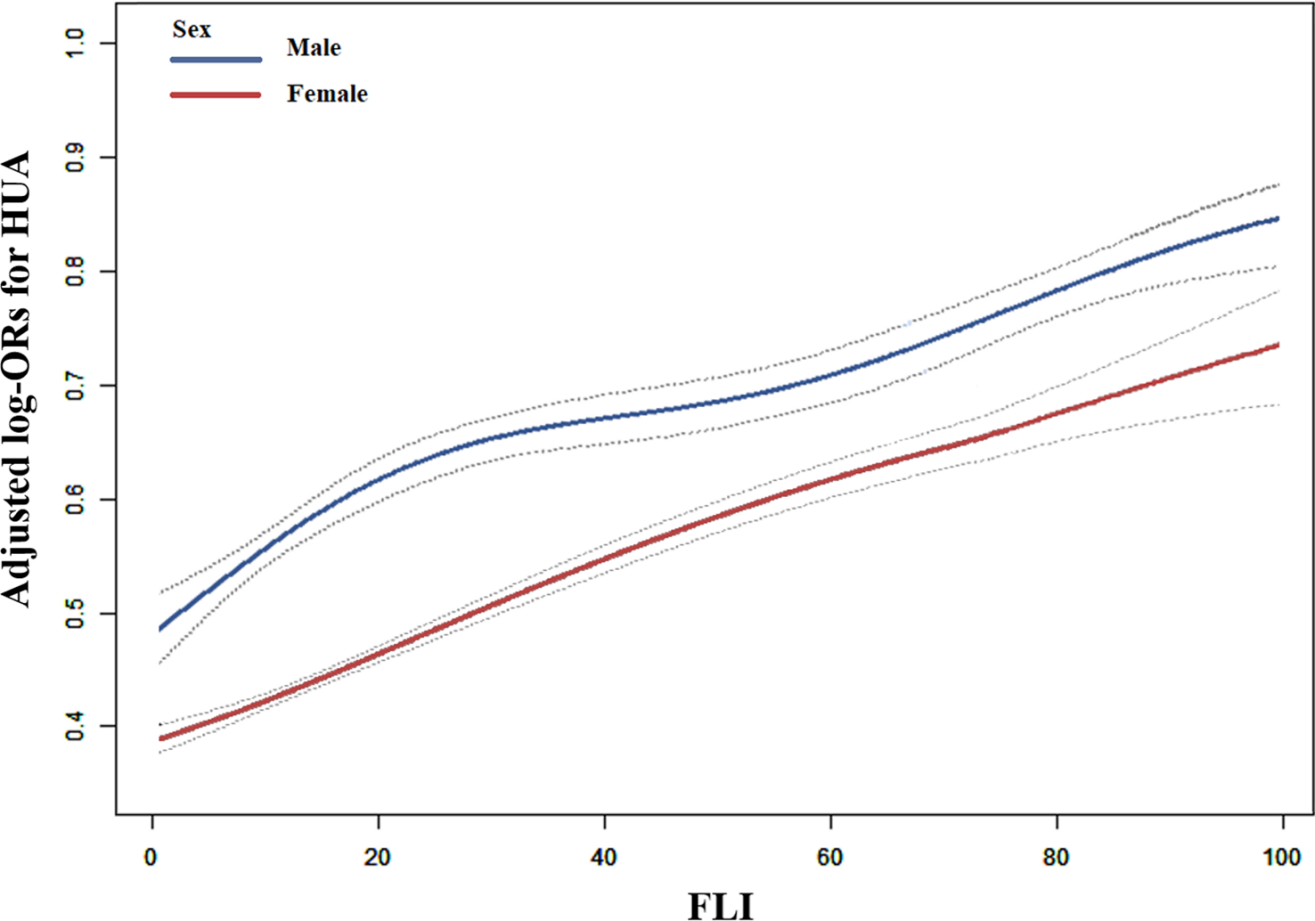
Correlation between FLI and HUA prevalence by sex in hypertensive Chinese patients. A linear correlation between FLI and HUA prevalence by sex was discovered (P < 0.05). The solid and dashed lines represent the estimated values in males and females, respectively. The adjustment factors included age, BMI, WC, Education, Living standard, Physical activity, current smoking, current drinking, Hcy, creatinine, eGFR, diabetes mellitus, antihypertensive drugs, and glucose-lowering drugs.

**Table 2** depicts logistic regression model results for the correlation between FLI and HUA. FLI and HUA had a significant positive correlation (**Figure 1**). Per each *SD* unit increase in FLI, the prevalence of HUA increased by 78% (OR, 1.78; 95% CI: 1.69–1.87). HUA prevalence (FLI < 30) was significantly higher in the steatosis group than in the normal group (FLI ≥ 30) after adjusting numerous confounding factors (OR, 2.53; 95% CI: 2.30–2.77). Additionally, we split the FLI into four quartiles for sensitivity analysis to further validate the results of this study. Finally, HUA prevalence gradually and significantly increased across all quartiles of FLI. We observed sex differences in the relationship between FLI (< 30 vs. ≥ 30) and HUA (P for interaction = 0.006); thus, we performed a sex stratification analysis. Both males and females exhibited a positive correlation between FLI and HUA prevalence. The correlation between FLI and HUA was stronger in females than in males (male: OR, 1.70; 95% CI: 1.58–1.83; female: 1.85; 95% CI: 1.73–1.98). The generalized additive model and fitted smoothing curve (penalized spline method) are consistent with multivariate logistic regression models for the different gender (**Figure 2**).

**Table 2:** Relative odds between FLI and HUA in different models among hypertensive patients in different sex.

FLI	Participants, *n*	Events, *N* (%)	HUA^b^, OR (95% CI) P-value			
			Crude	Model 1	Model 2	Model 3
All participants						
per SD increase	13716	6099 (44.5)	1.59 (1.53, 1.64) < 0.001	1.75 (1.68, 1.82) < 0.001	1.77 (1.69, 1.86) < 0.001	1.78 (1.69, 1.87) < 0.001
clinical cutoff						
< 30	7720	2792 (36.2)	1	1	1	1
≥ 30	5996	3307 (55.2)	2.17 (2.03, 2.33) < 0.001	2.54 (2.36, 2.74) < 0.001	2.56 (2.34, 2.81) < 0.001	2.53 (2.30, 2.77) < 0.001
Quartile^a^						
Q1 (< 10.4)	3429	1143 (33.3)	1	1	1	1
Q2 (10.4–24.7)	3429	1291 (37.6)	1.21 (1.09, 1.33) 0.0002	1.43 (1.29, 1.59) < 0.001	1.47 (1.30, 1.67) < 0.001	1.47 (1.30, 1.67) < 0.001
Q3 (24.7–48.7)	3429	1568 (45.7)	1.69 (1.53, 1.86) < 0.001	2.18 (1.96, 2.43) < 0.001	2.29 (2.01, 2.59) < 0.001	2.28 (2.01, 2.59) < 0.001
Q4 (≥ 48.7)	3429	2097 (61.2)	3.15 (2.85, 3.48) < 0.001	4.23 (3.79, 4.73) < 0.001	4.34 (3.80, 4.95) < 0.001	4.33 (3.78, 4.96) < 0.001
P for trend			< 0.001	< 0.001	< 0.001	< 0.001
Male						
per SD increase	6478	3954 (64.8)	1.56 (1.48, 1.65) <0.001	1.68 (1.58, 1.78) <0.001	1.64 (1.53, 1.76) <0.001	1.70 (1.58, 1.83) < 0.001
clinical cutoff						
< 30	3581	1906 (53.2)	1	1	1	1
≥ 30	2897	2048 (70.7)	2.12 (1.91, 2.35) < 0.001	2.28 (2.04, 2.54) < 0.001	2.14 (1.88, 2.45) < 0.001	2.22 (1.94, 2.55) < 0.001
Quartile						
Q1 (< 10.4)	1695	827 (48.8)	1	1	1	1
Q2 (10.4–24.7)	1509	851 (56.4)	1.36 (1.18, 1.56) < 0.001	1.43 (1.24, 1.64) < 0.001	1.53 (1.29, 1.81) < 0.001	1.56 (1.31, 1.84) < 0.001
Q3 (24.7–48.7)	1521	951 (62.5)	1.75 (1.52, 2.02) < 0.001	1.94 (1.68, 2.25) < 0.001	1.98 (1.66, 2.36) < 0.001	2.04 (1.71, 2.44) < 0.001
Q4 (≥ 48.7)	1753	1325 (75.6)	3.25 (2.81, 3.76) < 0.001	3.83 (3.28, 4.49) < 0.001	3.73 (3.09, 4.51) < 0.001	4.04 (3.33, 4.91) < 0.001
P for trend			< 0.001	< 0.001	< 0.001	< 0.001
Female						
per SD increase	7238	2145 (35.2)	1.69 (1.61, 1.79) < 0.001	1.80 (1.70, 1.90) < 0.001	1.89 (1.77, 2.02) < 0.001	1.85 (1.73, 1.98) < 0.001
clinical cutoff						
< 30	4139	886 (21.4)	1	1	1	1
≥ 30	3099	1259 (40.6)	2.51 (2.27, 2.79) < 0.001	2.75 (2.47, 3.06) < 0.001	2.94 (2.60, 3.34) < 0.001	2.80 (2.46, 3.18) < 0.001
Quartile						
Q1 (<10.4)	1734	316 (18.2)	1	1	1	1
Q2 (10.4–24.7)	1920	440 (22.9)	1.33 (1.13, 1.57) 0.0005	1.43 (1.22, 1.69) < 0.001	1.43 (1.18, 1.73) 0.0002	1.40 (1.16, 1.69) 0.0006
Q3 (24.7–48.7)	1908	617 (32.3)	2.14 (1.84, 2.50) < 0.001	2.41 (2.06, 2.82) < 0.001	2.58 (2.15, 3.10) < 0.001	2.48 (2.06, 2.99) < 0.001
Q4 (≥ 48.7)	1676	772 (46.1)	3.83 (3.28, 4.48) < 0.001	4.53 (3.86, 5.32) < 0.001	4.90 (4.05, 5.92) < 0.001	4.58 (3.77, 5.55) < 0.001
P for trend			< 0.001	< 0.001	<0.001	< 0.001
**P for interaction^c^**						**0.0063**

Crude was adjusted for none.

Model 1 was adjusted for age and sex (only for the overall population).

Model 2 was adjusted for age, sex (only for the overall population), BMI, WC, education, living standard, physical activity, current smoking, current drinking, Hcy, creatinine, and eGFR.

Model 3 was adjusted for age, sex (only for the overall population), BMI, WC, education, living standard, physical activity, current smoking, current drinking, Hcy, creatinine, eGFR, diabetes mellitus, antihypertensive drugs, glucose-lowering drugs.

FLI, Fatty Liver Index; HUA, hyperuricemia; CI, confidence interval

a Quartile grouped according to the ranges of FLI value in quartile were Quartile1 (0.3-10.4), Quartile2 (10.4-24.7), Quartile3 (24.7-48.7), and Quartile4 (48.7-100.0), respectively.

b HUA was defined as erum uric acid ≥ 360 μmol/L for females and ≥ 420 μmol/L for males.

c Test for interaction between Fatty Liver Index (< 30 vs. ≥ 30) and HUA (yes or no) among hypertensive patients in different sex.

### Stratified analyses by additional factors

**Figure 3:**
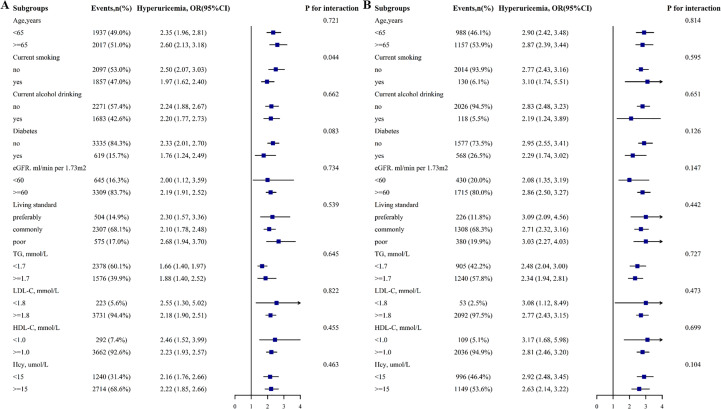
Stratified Analyses by Potential Modifiers of the Correlation between FLI and HUA prevalence by sex **(A)** males and **(B)** females. Each subgroup analysis adjusted for age, BMI, WC, education, living standard, physical activity, current smoking, alcohol intake, Hcy, creatinine, eGFR, diabetes mellitus, antihypertensive drugs, and glucose-lowering drugs.

A stratified analysis was conducted to examine further the relationship between FLI (< 30 vs. ≥ 30) and HUA among different subgroups (**Figure 3**). The results revealed that age (< 65 vs. ≥ 65 y), diabetes (no vs. yes), and other stratified variables did not significantly modify the relationship between FLI and HUA in different genders, except smoking in males (P-values for all interactions > 0.05).

## Discussion

This study discovered a strong positive correlation between FLI and HUA prevalence in hypertensive individuals, even after adjusting for various confounding factors. An additional evaluation of gender differences in the correlation between FLI and HUA prevalence revealed that the correlation was stronger in female subjects. This is the first known study of the strong positive correlation between FLI and HUA prevalence and the presence of sex differences in hypertensive individuals.

Previous studies have shown a synergistic effect between NAFLD and HUA (17, 36, 37). However, few reports have been published on the relationship between FLI and HUA. When Huang et al. (30) explored the relationship between visceral fat and HUA in the general population, they found that the risk of HUA increased significantly with FLI. In cellular and mouse models of NAFLD, increased expression of xanthine oxidase (XO), a rate-limiting enzyme that catalyzes uric acid production, could very well explain the underlying mechanisms of how NAFLD causes hyperuricemia (16). However, the sample size in Huang et al.’s (30) study is small (involving 1284 participants compared to 13,716 participants in the present study), leading to low statistical precision, less reliable, and less acceptable. Additionally, the study did not perform a sensitivity analysis, which would have cast doubt on the suitability of the results, or a subgroup analysis to explore potentially special subgroup of populations. This study compensates for these deficiencies.

This study is the first to report a sex difference in the correlation between FLI and HUA, suggesting that females may have a stronger correlation between FLI and HUA in hypertensive patients than males. The findings have been unreported in previous studies. The exact mechanism is unknown; possibly, hormonal differences underlie the underlying biochemical mechanisms. However, this may also be due to a study that included several postmenopausal females (18, 38, 39), who have significantly increased uric acid levels and NAFLD prevalence (40).

Previous studies have discovered that HUA can contribute to hypertension occurrence and development. The mechanism may involve decreasing vascular endothelial NO levels, oxidative stress, and activating the renin-angiotensin system (41, 42). Additionally, hypertension is a common and significantly elevated risk factor for HUA (43–45). The association between hyperuricemia and hypertension has been a subject of intense controversy (11, 43–48). Large-scale clinical trials are needed to determine if serum urate reduction can benefit hypertension and cardiometabolic disease. Simultaneously, hypertension and HUA are risk factors for multiple cardiovascular diseases (11, 49). The annual death toll from cardiovascular disease is as high as 18 million (50), Thus, early identification of high-risk groups of hypertensive patients with HUA is critical for the primary prevention of cardiovascular disease. According to this study, HUA prevalence in hypertension patients increased significantly with FLI. Therefore, FLI could be used as a useful predictor of NAFLD and to help optimize risk stratification management of HUA in hypertensive patients.

This study has the following advantages. According to our knowledge, this is the largest study to date to assess the correlation between FLI and HUA. Numerous confounding factors were adjusted, and group sensitivity analyses were performed to enhance the authenticity and reliability of this study (30, 51). Additionally, this study identified gender differences in the correlation between FLI and HUA. Several limitations of this study are also there. First, this cross-sectional study does not infer a causal relationship between FLI and HUA and needs further validation by large-scale prospective studies. Second, the study was conducted on Chinese hypertensive people, and the generalization to other populations remains to be verified. Third, this study lacks data on uric acid-lowering drugs (52–54). Fourth, heavy drinkers were included in the screening due to the lack of information on alcohol consumption in the questionnaire used for this study. This study aims to explore the relationship between NAFLD and HUA and assess the potential value of this index in the risk stratification of HUA in hypertensive individuals. Drinking status was adjusted as a confounding factor in the data analysis.

## Conclusions

This study demonstrated a significant positive correlation between FLI and HUA prevalence in hypertensive Chinese adults. Importantly, this positive correlation appears to be stronger in females. If further confirmed in clinical practice, FLI could be an important predictor tool for identifying NAFLD and to predict the risk of developing HUA in hypertensive patients. In summary, routine monitoring of FLI in hypertensive patients is recommended because FLI helps identify people at high risk of HUA. Hypertension and HUA are also major risk factors for cardiovascular complications.

## Data availability statement

The raw data supporting the conclusions of this article will be made available by the authors, without undue reservation.

## Ethics statement

This study was approved by the ethics committee of the Institute of Biomedicine, Anhui Medical University (NO.CH1059), and the Second Affiliated Hospital of Nanchang University (NO. 2018019). The patients/participants provided their written informed consent to participate in this study. All procedures performed in studies involving human participants followed the ethical standards of the institutional and national research committee and the 1964 Helsinki declaration and its later amendments or comparable ethical standards.

## Author contributions

CY participated in literature search, data analysis, and data interpretation. CY wrote the manuscript. XZ extracted and collected data. CY, XZ, WZ, TW, LZ, HB, and XC conceived of the study and participated in its design and coordination. WZ and LZ participated in the study design and provided critical revision. All authors contributed to the article and approved the submitted version.
