# Supplementary material for: Positive correlation between fatty liver index and hyperuricemia in hypertensive Chinese adults: a H-type hypertension registry study
Source: Front Endocrinol (Lausanne). 2023 Jun 2;14:1183666. doi: 10.3389/fendo.2023.1183666 (PMC10273275; doi:10.3389/fendo.2023.1183666)
Supplement: Supplementary file 1 [file DataSheet_1.docx]

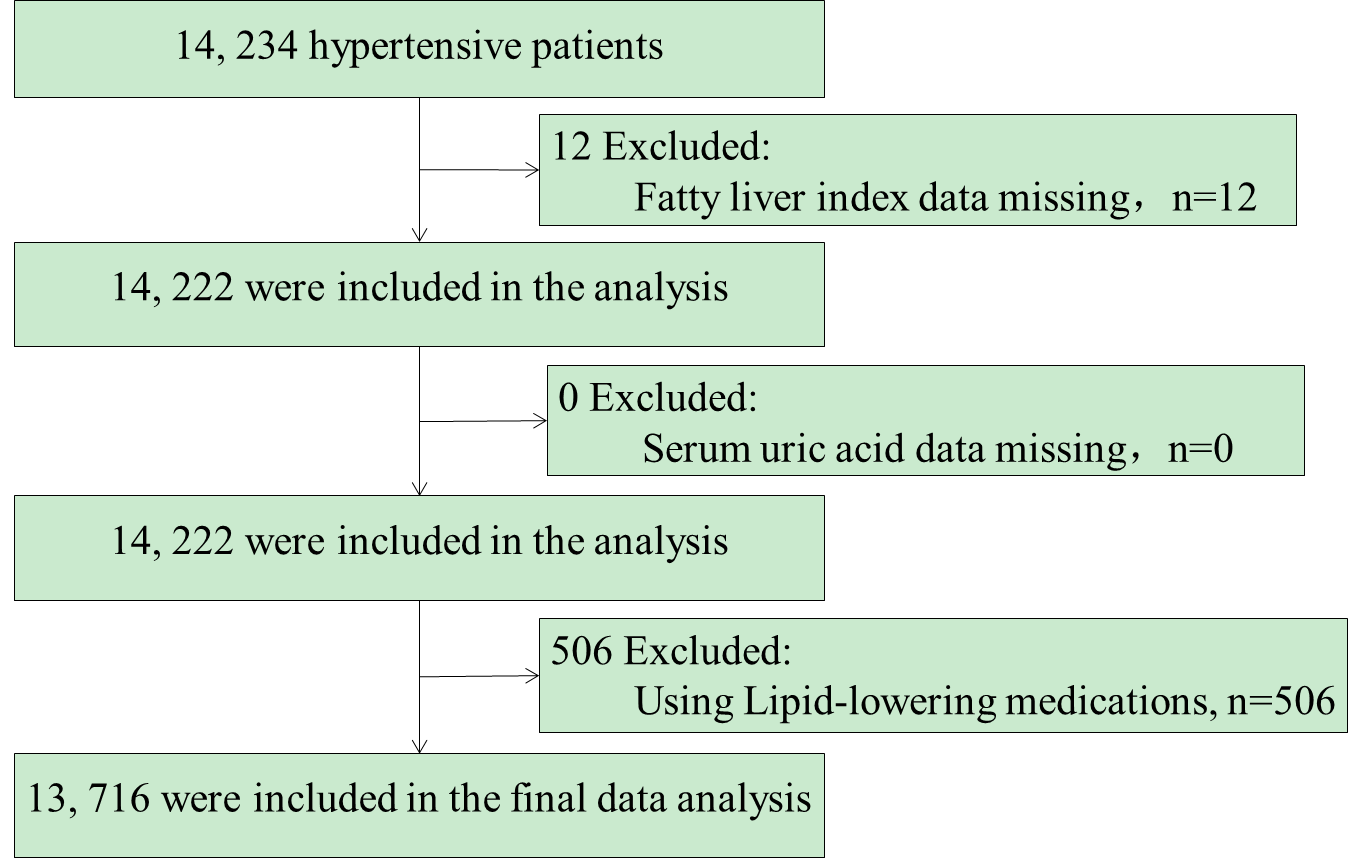


**Fig. S1. Flow diagram of the study participants.**

**Table S1. Baseline characteristics of the study participants according to FLI.**

| Characteristics | Total | FLI clinical cutoff | | P-value |
| --- | --- | --- | --- | --- |
|  |  | < 30 | ≥ 30 |  |
| Participants | 13716 | 7720 | 5996 |  |
| Age, year | 63.8 ± 9.4 | 65.8 ± 9.2 | 61.2 ± 9.0 | < 0.001 |
| Male, n (%) | 6478 (47.2) | 3581 (46.4) | 2897 (48.3) | 0.025 |
| BMI, kg/m^2^ | 23.6 ± 3.7 | 21.5 ± 2.5 | 26.3 ± 3.4 | < 0.001 |
| WC, cm | 83.7 ± 9.9 | 77.9 ± 7.2 | 91.3 ± 7.3 | < 0.001 |
| Education, n (%) |  |  |  | < 0.001 |
| Illiteracy | 4255 (38.3) | 2610 (41.6) | 1645 (34.1) |  |
| Primary | 4650 (41.9) | 2700 (43.0) | 1950 (40.4) |  |
| Secondary and above | 2197 (19.8) | 963 (15.4) | 1234 (25.6) |  |
| Living standard, n (%) |  |  |  | < 0.001 |
| preferably | 1456 (13.1) | 782 (12.5) | 674 (14.0) |  |
| commonly | 7458 (67.2) | 4167 (66.4) | 3291 (68.2) |  |
| poor | 2188 (19.7) | 1324 (21.1) | 864 (17.9) |  |
| Physical activity^a^, n (%) |  |  |  | 0.026 |
| Mild | 6203 (55.9) | 3457 (55.1) | 2746 (56.9) |  |
| Moderate | 2589 (23.3) | 1454 (23.2) | 1135 (23.5) |  |
| Vigorous | 2310 (20.8) | 1362 (21.7) | 948 (19.6) |  |
| Current smoking, n (%) | 3564 (26.0) | 2104 (27.3) | 1460 (24.4) | < 0.001 |
| Current alcohol drinking, n (%) | 3005 (21.9) | 1554 (20.1) | 1451 (24.2) | < 0.001 |
| GGT, U/L | 33.2 ± 43.2 | 21.2 ± 18.1 | 48.5 ± 58.5 | < 0.001 |
| TC, mmol/L | 5.2 ± 1.1 | 5.0 ± 1.0 | 5.4 ± 1.1 | < 0.001 |
| TG, mmol/L | 1.8 ± 1.2 | 1.3 ± 0.6 | 2.5 ± 1.5 | < 0.001 |
| LDL-C, mmol/L | 3.0 ± 0.8 | 2.8 ± 0.8 | 3.2 ± 0.8 | < 0.001 |
| HDL-C, mmol/L | 1.6 ± 0.4 | 1.7 ± 0.4 | 1.4 ± 0.4 | < 0.001 |
| Hcy, μmol/L | 18.0 ± 11.0 | 18.1 ± 10.6 | 17.8 ± 11.5 | < 0.001 |
| Creatinine, μmol/L | 72.7 ± 46.4 | 73.3 ± 52.2 | 71.9 ± 37.7 | < 0.001 |
| eGFR, mL/min/1.73 m^2^ | 88.3 ± 20.2 | 87.1 ± 20.4 | 89.9 ± 19.8 | < 0.001 |
| Serum urate, μmol/L | 419.1 ± 120.7 | 396.0 ± 114.0 | 448.9 ± 122.6 | < 0.001 |
| Hyperuricemia, n (%) | 6099 (44.5) | 2792 (36.2) | 3307 (55.2) | < 0.001 |
| Diabetes mellitus^b^, n (%) | 2436 (17.8) | 898 (11.6) | 1538 (25.7) | < 0.001 |
| Antihypertensive drugs, n (%) | 8781 (64.0) | 4808 (62.3) | 3973 (66.3) | < 0.001 |
| Glucose-lowering drugs, n (%) | 661 (4.8) | 239 (3.1) | 422 (7.0) | < 0.001 |
| FLI | 31.8 ± 25.1 | 13.1 ± 7.9 | 55.8 ± 18.1 | < 0.001 |

Abbreviation: *FLI*, Fatty Liver Index; *BMI*, body mass index; *WC*, waist circumference; *DBP*, diastolic blood pressure; *GGT*, glutamyltransferase; *TC*, total cholesterol; *TG*, triglycerides; *HDL-C*, high-density lipoprotein cholesterol; *LDL-C*, low-density lipoprotein cholesterol; *Hcy*, homocysteine; *eGFR*, estimated glomerular filtration rate.

^a^Physical activity was defined as mild, moderate, or vigorous according to the participant’s personal evaluation.

^b^diabetes mellitus was defined as self-reported physician diagnosis of diabetes or FBG concentration ≥ 7.0 mmol/L or use of glucose-lowering drugs.
